# *Mycobacterium avium *subspecies *paratuberculosis*, Crohn's disease and the Doomsday scenario

**DOI:** 10.1186/1757-4749-1-15

**Published:** 2009-07-14

**Authors:** John Hermon-Taylor

**Affiliations:** 1 Division of Nutritional Sciences, Franklin-Wilkins Building, King's College London, 150 Stamford Street, London, SE1 9NH, UK

## Abstract

Johne's disease is chronic inflammation of the intestine caused by *Mycobacterium avium *subspecies *paratuberculosis*. Infection and disease are mainly in domestic livestock but can affect many species including primates. Johne's is a new disease which emerged at the turn of the 19^th ^and 20^th ^centuries and principally involved Europe and North America. It has since spread to former low incidence regions to become a global problem. Crohn's disease is a chronic inflammation of the intestine in humans which emerged in Europe and North America mid 20^th ^century and increased to become a major healthcare problem. It has now spread to former low incidence regions. Infected animals shed *Mycobacterium avium *subspecies *paratuberculosis *in milk and into the environment. Human populations are widely exposed. Outcomes maybe influenced by microbial phenotype. Exposure to extracellular forms of these pathogens may confer some natural protection; exposure to intracellular forms which have passaged through milk macrophages or environmental protists may pose a greater threat to humans particularly individuals with an inherited or acquired susceptibility. Hot spots of human disease such as in Winnipeg which sits on rock at the junction of two rivers may result from local exposure to high levels of waterborne pathogens brought down from farmland. When appropriate methods are used most people with Crohn's disease are found to be infected. There are no data which demonstrate that these pathogens are harmless to humans. An overwhelming balance of probability and Public health risk favours the conclusion that *Mycobacterium avium *subspecies *paratuberculosis *is also pathogenic for people. A two tier co-operative pathogenic mechanism is proposed in Crohn's disease. Intracellular infection with the primary pathogen widely distributed throughout the gut causes an immune dysregulation and a specific chronic enteric neuropathy with loss of mucosal integrity. Segments of gross inflammatory disease result from the perturbed neuroimmune response to penetration into the gut wall of secondary pathogens from the lumen. These include both normal gut organisms and educated members of the enteric microbiome such as more aggressive *E. coli*. More new diseases may arise from failure to apply a range of remedial measures to this longstanding zoonotic problem.

## Review

### MAP and the emergence of Johne's and Crohn's diseases

Johne's disease (JD) is a systemic infection and chronic inflammation of the intestine in animals caused by *Mycobacterium avium *subspecies *paratuberculosis *(MAP). It is most common in ruminants but can affect many other species including primates. It was first seen to emerge in Europe and North America at the end of the 19^th ^and beginning of the 20^th ^centuries. Crohn's disease (CD) in humans is a systemic disorder whose principal clinico-pathological manifestation is also chronic inflammation of the intestine. It is also a new disease which was first seen to emerge in the same continents 40–50 years after JD and increased in frequency steadily until it has become a major healthcare problem. In some areas in the USA in recent years the incidence of CD has seemed to plateau around 7–8 per 10^5 ^population per year [1,2]. In Europe the incidence of CD in adults continues to grow [3-5]. Studies in Stockholm, Czech Republic, and Australia supported by data from Finland suggest that the incidence of CD in children in these areas in recent years has been rising in some cases as high as about 5 fold per decade [6-9]. These rapid changes in incidence rule out a primary genetic causation of CD. The data from recent genome wide scans which has identified 32 significant genomic loci related to susceptibility to CD are consistent with the involvement of intracellular bacterial pathogens including mycobacteria, in disease causation [10].

The rising incidence of CD reported from several former low incidence countries in Asia shows that, as with JD, CD is spreading worldwide [11,12]. Recent work from New Zealand reported a high incidence of CD of 16.5 per 10^5 ^per year affecting the Canterbury region of South Island with Christchurch as its principal city [13]. Mountains are to the northwest and rivers from them run across rich agricultural pastures and either side of Christchurch before entering the sea. A small river meanders through the city itself. Some of these features are reminiscent of the situation in Cardiff, South Wales UK where a high incidence of CD in city wards bordering the river Taff draining the upland pastures of the Brecons and running through the city was consistent with exposure of the local population to aerosols from the river [14].

A conspicuously anomalous distribution in the incidence of CD exists in North America either side of the Canadian border between Minnesota and Manitoba. In Minnesota to the south the well documented population-based incidence of CD in Olmsted County is 7.9 per 10^5^/year [1] whereas in some areas of the city of Winnipeg little more than 400 miles to the north the incidence reaches a maximum 3.5 fold greater at 28.07 per 10^5^/year [15]. Winnipeg lies astride the junction of the Red River of the North running up from the south and the Assiniboine River coming in from the west. The city sits on bedrock which was once the floor of the immense prehistoric glacial lake Agassiz, with scant run-off in permeable sand and gravel aquifers [16]. The 'hot spot' of CD in the city of Winnipeg we see now is probably due to local exposure of the human population to high levels of waterborne MAP brought down from the agricultural river catchments of the US Midwest, meeting those from the provinces of Manitoba, Saskatchewan and Alberta. Waterborne MAP under these conditions would almost certainly include organisms which have adopted the intracellular phenotype having been taken up by abundant environmental protists [17].

### Movement of people and pathogen

Migrant studies show that the incidence of CD in people moving from a low CD and JD incidence area to a high incidence area subsequently rises to that of the host population. The inverse situation is that in which MAP is introduced into an isolated community usually by importation of infected animals. This happened in Iceland in 1933 [reviewed in [18]]. After a latent period following introduction of the pathogen there were at intervals successive epidemics of JD in the island sheep, then in the cattle, then CD in the human population. From 1960 to a peak in 1992 the incidence of CD increased 18 fold. Thus in either case, if people move in amongst MAP or if MAP is moved in amongst people the result is the same namely a steep rise in the incidence of CD. The time interval between the emergence and rise of JD in animals and CD in humans in Iceland was again about 40–50 years. With the almost unlimited opportunity for MAP to spread and evolve in intensively farmed domestic livestock and associated contaminated environments over more than a half century, an evolving virulence and species adaptation of the pathogen would be reflected in the JD to CD interval becoming shorter. A recent example of this happening maybe the steep 4.5 fold increase in CD in the Czech Republic 1995–2007 following the rise in JD caused by the unimpeded importation of subclinically infected cattle from Western Europe after independence in 1990.

### Natural Immunity to MAP from environmental and occupational exposure

Why don't dairy farmers and veterinarians exposed to MAP-infected animals get a much higher incidence of CD? Data from the US show that these occupations are in fact associated with a significantly reduced death rate from Inflammatory Bowel Disease [19]. Children exposed to farm animals, particularly cattle, in early life also subsequently have a lower incidence of CD [20]. Occupational exposure to MAP is associated with raised antibody levels to MAP lysates [21]. An answer to the question consistent with these observations is that the extracellular classical ZN-positive phenotype of MAP excreted in trillions by heavily infected animals is not one to which humans are most susceptible. Exposure to this form of the organism may result in the acquisition of some natural immunity to disease. A good example of this happening as a result of purposeful exposure is the approximate 10 fold reduction in clinical Johne's disease achieved subsequently by vaccination of calves using conventional whole killed MAP vaccines with the organisms in this form [22]. The well described urban preponderance of CD may not be that townsfolk have an increased susceptibility to CD, rather that country folk have some natural protection. Passage of MAP through bovine macrophages in milk and cheese or through environmental protists would result in a switch to an intracellular phenotype of MAP likely to have an enhanced virulence for humans [23,24].

### MAP and the convergence of candidate pathogens

With the new 21^st ^century, a steadily increasing volume of parallel research has identified three principal sets of bacteria as candidates for the causation of the gross inflammatory disease of the intestine in CD. These are the community of normal gut flora [25], abnormal gut flora such as adherent invasive *E. coli *(AIEC) [26], and MAP. Because of the global advance of CD and the serious implications for Public Health as well as cumulative individual suffering, there is a need for researchers and clinicians in the field to recognise that the reliable evidence obtained from each of the three lines of inquiry is convergent and that there is actually no conflict between them.

From experimental as well as clinical evidence there is no doubt that bacteria from the normal intestinal microbial community can infect and inflame the gut wall and that they do so in CD. However, the spontaneous emergence and rise of CD in human populations across the globe due to an epidemic of normal gut flora, in the absence of another specific initiating cause, seems rather improbable. The enteric microbiome is a fertile environment for horizontal gene transfer [27]. Advancement of pathogenicity in bacteria may follow the acquisition and mutation of genes and changes in their regulation [28-30]. We already have examples of the pathological consequences of such adaptation in common gut bacteria such as *E. coli *which can be enteropathogenic, enterohaemorrhagic, enterotoxigenic, enteroaggregative and recently enteroadherent and invasive AIEC [31]. Such adaptations usually arise due to the imposition of some external selection pressure. Recent evidence also suggests that common enteric bacteria like *E. coli *may display predictive behaviour [32].

The principal property of MAP which distinguishes it from all other candidate pathogens in the primary causation of CD is that it is an established multi-host chronic enteric pathogen. MAP has the proven specific ability to initiate and maintain chronic inflammation of the intestine of a range of different histopathological types in many species including primates. MAP infection in animals causes a local and systemic immune dysregulation. It is also specifically neuropathogenic especially for non-myelinated neurones and intestinal disease is accompanied by a chronic enteric neuropathy [33]. Despite its broad pathogenicity, MAP infection can persist in animals for years without necessarily progressing to clinical disease. Clinical disease in animals when it occurs is commonly of the pluribacillary type but paucimicrobial disease with the pathogens in a Ziehl Neelsen (ZN)-negative phenotype is well described.

The overall prevalence of MAP infection in US dairy herds is reported by a USDA survey to be 68.1% [34]. A range of broadly similar data shows that MAP infection in farm animals is widespread in many areas of Western Europe and elsewhere. MAP contaminates and persists in water and the environment, is in dairy products, can survive milk pasteurisation, and is present in meat from infected animals. It is inevitable that human populations are widely exposed.

### MAP in humans

MAP infection in humans is difficult to detect. The organisms are present in low abundance in a robust ZN-negative phenotype. They are intracellular and minimise their own immune recognition. They are extremely difficult to isolate and propagate in culture and are relatively resistant to chemical and enzymatic lysis. Reliable access to their DNA is only achieved during sample processing by combining exposure to stringent lysis buffers with an additional optimised mechanical disruption step. Freezing samples and tissue extracts especially at -20°C substantially reduces the PCR detection rate of their GC-rich DNA. The organisms have been cultured and detected in blood showing that, as in animals, the infection in humans is systemic [35-37]. At present, the benchmark diagnostic test for MAP infection in humans is nested PCR applied to single ~20 mg fresh endoscopic mucosal biopsies [38]. When validated methodologies have been used most people with CD have been found to be infected with MAP [39]. In simple words, most people with chronic inflammation of the intestine (of the CD type) are infected with a mycobacterium which is a proven specific cause of chronic inflammation of the intestine. There are no data which demonstrate that MAP are harmless to humans. The overwhelming balance of probability and public health risk favours the conclusion that MAP are also pathogenic for people.

### Inflammation in Crohn's disease caused by a two tier co-operative pathogenic mechanism

MAP infects the gut widely in CD and is found both in the more normal looking intestine and the grossly inflamed and diseased segments of intestine [33]. MAP antigens have appeared to dominate the immunological responses of intestinal CD4 T cell lines from patients with CD [40]. Mannans released by MAP inhibit intracellular killing of internalised bacteria [41].

The MAP infection causes a *primary *microscopic inflammation accompanied by a specific immune dysregulation and enteric neuropathy [33]. Mucosal integrity and other critical functions of the intestine are impaired. The visible segments of gross inflammatory disease result from the perturbed neuroimmune response to the *secondary *penetration into the gut wall of gut flora containing both normal intestinal bacteria and those which have undergone transformations leading to a more invasive phenotype like AIEC. It is important to note that genomic loci in the host conferring genetic susceptibility to Crohn's disease have the potential to operate at the levels of both primary and secondary pathogens. The entry of food residues into the gut wall contributes an allergic component to the inflammatory mess. Although MAP has been found in intestinal granulomas in humans [42], the presence or absence of these and other features of the variable histopathological picture of CD are principally determined by the large scale response to the secondary co-pathogens including especially other granulomatous species like *M. avium *subspecies *avium *which are frequently recovered in culture from CD tissues [38]. Thus the three lines of contemporary research inquiry come together in a two tiered co-operative pathogenic mechanism.

### MAP doomsday

Imagine the collective human enteric microbiome in, say, a crowded Europe. A vast composite structure made up of millions of individual highly mobile microbial reservoirs variably interconnected in time and space and degree. A dynamic structure possessing an inherent self governing order and stability not easily displaced. Into this cellular system is progressively introduced a slowly growing specific mycobacterial pathogen which has acquired the genetic machinery necessary to cloak itself with a predicted fucosylated surface [43] so that it conforms with the familiar molecular environment particularly of the host's epithelial cells and mucosal compartment [44]. It has come from the parallel universe of the collective enteric microbiome of human food animals and before that from the soil. It causes a microscopic inflammation and perturbs the microenvironment of the mucosa and gut wall. To survive and prosper it minimises its confrontation with the human immune system. It causes a variable immune dysregulation but it also inflames the fine structure and function of the enteric nervous system.

More than a hundred years go by. Both animal and human total microbiomes swell with increasing population density. The mycobacterial pathogen acquires additional properties resulting in an evolution in its behaviour with an increase in pathogenicity and species range. Some normal inhabitants of the enteric microbiome adapt to the disturbed intestinal microenvironment and they too acquire characteristics which make them more invasive. Chronic enteric disease emerges and spreads particularly in individuals with an inherited or acquired susceptibility. Humans responsible for controlling and managing these diseases, blind to what is really happening are distracted by detail and dismissive. The required remedial measures are not designed and applied and the problems get worse. Left undisturbed, maybe the education in hostility already received by increasingly aggressive members of the former normal gut flora will progress to the point where they too can emerge from background to become primary independent pathogens in their own right. When they do so more new diseases will emerge.

### Can anti-MAP treatment heal Crohn's disease?

The answer to this question supported by a correct interpretation of data both from open label studies [45-48] and the Australian controlled clinical trial is a qualified yes [49-51]. It can in some people with CD some of the time. When it does so in 'responders' receiving treatment with drug combinations including rifabutin and clarithromycin the clinical and pathological improvement can be dramatic and has been associated with the conversion of pre-treatment MAP positive tests in blood [52] and gut mucosa (my own unpublished observations) to negative. Furthermore, some of the clinical benefit resulting from treatment of CD with conventional 'immunosuppressive' agents such as 6-mercaptopurine or methotrexate may actually be a consequence of their demonstrable direct anti-MAP action [53-55]. But MAP infections are difficult to eradicate. The organisms are generally resistant in vivo to drugs conventionally used in the treatment of tuberculosis. Treatment is prone to all the problems of microbial drug resistance and latency encountered in the management of chronic lung disease caused by other members of the *M. Avium *Complex.

New clinical trials are needed of anti-MAP treatment in CD particularly of agents developed for the treatment of *M. tuberculosis *which are active against mycobacteria in the non-replicative state [56] and where the gene encoding the molecular target is shared by MAP. Rich clinical and commercial rewards are out there for those who do so successfully.

## Conclusion

Recognition and acceptance of the true nature of the expanding long term threat to human health posed by widespread exposure to MAP, based upon a perceptive understanding of the problem and the overwhelming balance of reliable scientific evidence, is a matter of urgency. The solutions lie in the identification and incremental introduction of a range of remedial measures which are both scientific and regulatory whose effective application on a global scale requires close international cooperation.

## Abbreviations

MAP: *Mycobacterium avium *subspecies *paratuberculosis*; JD: Johne's disease; CD: Crohn's disease; AIEC: adherent Invasive *E. coli*; ZN: Ziehl Neelsen; US: United States of America; USDA: US Department of Agriculture.

## Competing interests

The author currently owns the patents to a virally vectored vaccine against *Mycobacterium avium *subspecies *paratuberculosis *intended as a treatment for MAP infection in humans.

## Authors' contributions

I confirm that this is my work.

## References

[B1] Loftus CG, Loftus EV, Harmsen WS, Zinsmeister AR, Tremaine WJ, Melton LJ, Sandborn WJ (2007). Update on the incidence and prevalence of Crohn's disease and ulcerative colitis in Olmsted County, Minnesota, 1940–2000. Inflamm Bowel Dis.

[B2] Herrinton LJ, Liu L, Lewis JD, Griffin PM, Allison J (2008). Incidence and prevalence of inflammatory bowel disease in a Northern California managed care organization, 1996–2002. Am J Gastroenterol.

[B3] Molinié F, Gower-Rousseau C, Yzet T, Merle V, Grandbastien B, Marti R, Lerebours E, Dupas JL, Colombel JF, Salomez JL, Cortot A (2004). Opposite evolution in incidence of Crohn's disease and ulcerative colitis in Northern France (1988–1999). Gut.

[B4] Lapidus A (2006). Crohn's disease in Stockholm County during 1990–2001: an epidemiological update. World J Gastroenterol.

[B5] Vind I, Riis L, Jess T, Knudsen E, Pedersen N, Elkjaer M, Bak Andersen I, Wewer V, Nørregaard P, Moesgaard F, Bendtsen F, Munkholm P, DCCD study group (2006). Increasing incidences of inflammatory bowel disease and decreasing surgery rates in Copenhagen City and County, 2003–2005: a population-based study from the Danish Crohn colitis database. Am J Gastroenterol.

[B6] Hildebrand H, Finkel Y, Grahnquist L, Lindholm J, Ekbom A, Askling J (2003). Changing pattern of paediatric inflammatory bowel disease in northern Stockholm 1990–2001. Gut.

[B7] Pozler O, Maly J, Bonova O, Dedek P, Frühauf P, Havlickova A, Janatova T, Jimramovsky F, Klimova L, Klusacek D, Kocourkova D, Kolek A, Kotalova R, Marx D, Nevoral J, Petro R, Petru O, Plasilova I, Seidl Z, Sekyrova I, Semendak N, Schreierova I, Stanek J, Sykora J, Sulakova A, Toukalkova L, Travnickova R, Volf V, Zahradnicek L, Zenisková I (2006). Incidence of Crohn disease in the Czech Republic in the years 1990 to 2001 and assessment of pediatric population with inflammatory bowel disease. J Pediatr Gastroenterol Nutr.

[B8] Phavichitr N, Cameron DJ, Catto-Smith AG (2003). Increasing incidence of Crohn's disease in Victorian children. J Gastroenterol Hepatol.

[B9] Turunen P, Kolho KL, Auvinen A, Iltanen S, Huhtala H, Ashorn M (2006). Incidence of inflammatory bowel disease in Finnish children, 1987–2003. Inflamm Bowel Dis.

[B10] Barrett JC, Hansoul S, Nicolae DL, Cho JH, Duerr RH, Rioux JD, Brant SR, Silverberg MS, Taylor KD, Barmada MM, Bitton A, Dassopoulos T, Datta LW, Green T, Griffiths AM, Kistner EO, Murtha MT, Regueiro MD, Rotter JI, Schumm LP, Steinhart AH, Targan SR, Xavier RJ, Libioulle C, Sandor C, Lathrop M, Belaiche J, Dewit O, Gut I, Heath S, Laukens D, Mni M, Rutgeerts P, Van Gossum A, Zelenika D, Franchimont D, Hugot JP, de Vos M, Vermeire S, Louis E, Cardon LR, Anderson CA, Drummond H, Nimmo E, Ahmad T, Prescott NJ, Onnie CM, Fisher SA, Marchini J, Ghori J, Bumpstead S, Gwilliam R, Tremelling M, Deloukas P, Mansfield J, Jewell D, Satsangi J, Mathew CG, Parkes M, Georges M, Daly MJ, NIDDK IBD Genetics Consortium, Belgian-French IBD Consortium, Wellcome Trust Case Control Consortium (2008). Genome-wide association defines more than 30 distinct susceptibility loci for Crohn's disease. Nat Genet.

[B11] Thia KT, Loftus EV, Sandborn WJ, Yang SK (2008). An update on the epidemiology of inflammatory bowel disease in Asia. Am J Gastroenterol.

[B12] Leong RW, Lau JY, Sung JJ (2004). The epidemiology and phenotype of Crohn's disease in the Chinese population. Inflamm Bowel Dis.

[B13] Gearry RB, Richardson A, Frampton CM, Collett JA, Burt MJ, Chapman BA, Barclay ML (2006). High incidence of Crohn's disease in Canterbury, New Zealand: results of an epidemiologic study. Inflamm Bowel Dis.

[B14] Pickup RW, Rhodes G, Arnott S, Sidi-Boumedine K, Bull TJ, Weightman A, Hurley M, Hermon-Taylor J (2005). *Mycobacterium avium *subsp. *paratuberculosis *in the catchment area and water of the River Taff in South Wales, United Kingdom, and its potential relationship to clustering of Crohn's disease cases in the city of Cardiff. Appl Environ Microbiol.

[B15] Green C, Elliott L, Beaudoin C, Bernstein CN (2006). A population-based ecologic study of inflammatory bowel disease: searching for etiologic clues. Am J Epidemiol.

[B16] Province of Manitoba Department of Natural Resources, Water Resources Branch. Aquifer maps of southern Manitoba; map 2: Sand and Gravel Aquifers. http://www.manitoba.ca/waterstewardship/floodinfo/maps/images/sand_gravel_aquifers.jpg.

[B17] Mura M, Bull TJ, Evans H, Sidi-Boumedine K, McMinn L, Rhodes G, Pickup R, Hermon-Taylor J (2006). Replication and long-term persistence of bovine and human strains of *Mycobacterium avium *subsp. *paratuberculosis *within Acanthamoeba polyphaga. Appl Environ Microbiol.

[B18] Hermon-Taylor J, El-Zaatari FAK, Pedley S, Bartram J, Rees G, Dufour A, Cotruvo JA (2004). The *Mycobacterium avium *subspecies *paratuberculosis *problem and its relation to the causation of Crohn disease. Pathogenic Mycobacteria in Water: a guide to Public Health Consequences, Monitoring and Management.

[B19] Cucino C, Sonnenberg A (2001). Occupational mortality from inflammatory bowel disease in the United States 1991–1996. Am J Gastroenterol.

[B20] Radon K, Windstetter D, Poluda AL, Mueller B, von Mutius E, Koletzko S, Chronische Autoimmunerkrankungen und Kontakt zu Tieren (Chronic Autoimmune Disease and Animal Contact) Study Group (2007). Contact with farm animals in early life and juvenile inflammatory bowel disease: a case-control study. Pediatrics.

[B21] Chiodini RJ, Thayer WR, Coutu JA, Chiodini RJ, Hines ME, Collins MT (1996). Presence of *Mycobacterium paratuberculosis *antibodies in animal healthcare workers. Proceedings of the Fifth International Colloquium on Paratuberculosis 1996.

[B22] Rosseels V, Huygen K (2008). Vaccination against paratuberculosis. Expert Rev Vaccines.

[B23] Cirillo JD, Falkow S, Tompkins LS, Bermudez LE (1997). Interaction of *Mycobacterium avium *with environmental amoebae enhances virulence. Infect Immun.

[B24] Patel D, Danelishvili L, Yamazaki Y, Alonso M, Paustian ML, Bannantine JP, Meunier-Goddik L, Bermudez LE (2006). The ability of *Mycobacterium avium *subsp. *paratuberculosis *to enter bovine epithelial cells is influenced by preexposure to a hyperosmolar environment and intracellular passage in bovine mammary epithelial cells. Infect Immun.

[B25] Macfarlane GT, Blackett KL, Nakayama T, Steed H, Macfarlane S (2009). The gut microbiota in inflammatory bowel disease. Curr Pharm Des.

[B26] Martinez-Medina M, Aldeguer X, Lopez-Siles M, González-Huix F, López-Oliu C, Dahbi G, Blanco JE, Blanco J, Garcia-Gil LJ, Darfeuille-Michaud A (2009). Molecular diversity of *Escherichia coli *in the human gut: new ecological evidence supporting the role of adherent-invasive *E. coli *(AIEC) in Crohn's disease. Inflamm Bowel Dis.

[B27] Kurokawa K, Itoh T, Kuwahara T, Oshima K, Toh H, Toyoda A, Takami H, Morita H, Sharma VK, Srivastava TP, Taylor TD, Noguchi H, Mori H, Ogura Y, Ehrlich DS, Itoh K, Takagi T, Sakaki Y, Hayashi T, Hattori M (2007). Comparative metagenomics revealed commonly enriched gene sets in human gut microbiomes. DNA Res.

[B28] Ahmed N, Dobrindt U, Hacker J, Hasnain SE (2008). Genomic fluidity and pathogenic bacteria: applications in diagnostics, epidemiology and intervention. Nat Rev Microbiol.

[B29] Perfeito L, Fernandes L, Mota C, Gordo I (2007). Adaptive mutations in bacteria: high rate and small effects. Science.

[B30] Osborne SE, Walthers D, Tomljenovic AM, Mulder DT, Silphaduang U, Duong N, Lowden MJ, Wickham ME, Waller RF, Kenney LJ, Coombes BK (2009). Pathogenic adaptation of intracellular bacteria by rewiring a cis-regulatory input function. Proc Natl Acad Sci USA.

[B31] Darfeuille-Michaud A, Neut C, Barnich N, Lederman E, Di Martino P, Desreumaux P, Gambiez L, Joly B, Cortot A, Colombel JF (1998). Presence of adherent *Escherichia coli *strains in ileal mucosa of patients with Crohn's disease. Gastroenterology.

[B32] Tagkopoulos I, Liu YC, Tavazoie S (2008). Predictive behaviour within microbial genetic networks. Science.

[B33] Scanu AM, Bull TJ, Cannas S, Sanderson JD, Sechi LA, Dettori G, Zanetti S, Hermon-Taylor J (2007). *Mycobacterium avium *subspecies *paratuberculosis *infection in cases of irritable bowel syndrome and comparison with Crohn's disease and Johne's disease: common neural and immune pathogenicities. J Clin Microbiol.

[B34] Johne's Disease on U.S. Dairies, 1991–2007 (info sheet, 4p, 4/08). http://nahms.aphis.usda.gov.

[B35] Naser SA, Ghobrial G, Romero C, Valentine JF (2004). Culture of *Mycobacterium avium *subspecies *paratuberculosis *from the blood of patients with Crohn's disease. Lancet.

[B36] Bentley RW, Keenan JI, Gearry RB, Kennedy MA, Barclay ML, Roberts RL (2008). Incidence of *Mycobacterium avium *subspecies *paratuberculosis *in a population-based cohort of patients with Crohn's disease and control subjects. Am J Gastroenterol.

[B37] Kirkwood CD, Wagner J, Boniface K, Vaughan J, Michalski WP, Catto-Smith AG, Cameron DJ, Bishop RF (2009). *Mycobacterium avium *subspecies *paratuberculosis *in children with early-onset Crohn's disease. Inflamm Bowel Dis.

[B38] Bull TJ, McMinn EJ, Sidi-Boumedine K, Skull A, Durkin D, Neild P, Rhodes G, Pickup R, Hermon-Taylor J (2003). Detection and verification of *Mycobacterium avium *subsp.*paratuberculosis *in fresh ileocolonic mucosal biopsy specimens from individuals with and without Crohn's disease. J Clin Microbiol.

[B39] Feller M, Huwiler K, Stephan R, Altpeter E, Shang A, Furrer H, Pfyffer GE, Jemmi T, Baumgartner A, Egger M (2007). *Mycobacterium avium *subspecies *paratuberculosis *and Crohn's disease: a systematic review and meta-analysis. Lancet Infect Dis.

[B40] Olsen I, Tollefsen S, Aagaard C, Reitan LJ, Bannantine JP, Andersen P, Sollid LM, Lundin KE (2009). Isolation of *Mycobacterium avium *subspecies *paratuberculosis *reactive CD4 T cells from intestinal biopsies of Crohn's disease patients. PLoS One.

[B41] Mpofu CM, Campbell BJ, Subramanian S, Marshall-Clarke S, Hart CA, Cross A, Roberts CL, McGoldrick A, Edwards SW, Rhodes JM (2007). Microbial mannan inhibits bacterial killing by macrophages: a possible pathogenic mechanism for Crohn's disease. Gastroenterology.

[B42] Ryan P, Aarons S, Bennett MW, Lee G, O'Sullivan GC, O'Connell J, Shanahan F (2002). *Mycobacterium paratuberculosis *detected by nested PCR in intestinal granulomas isolated by LCM in cases of Crohn's disease. Methods Mol Biol.

[B43] Sheridan JM, Bull TJ, Hermon-Taylor J (2003). Use of bioinformatics to predict a function for the GS element in *Mycobacterium avium *subspecies *paratuberculosis*. J Mol Microbiol Biotechnol.

[B44] Coyne MJ, Reinap B, Lee MM, Comstock LE (2005). Human symbionts use a host-like pathway for surface fucosylation. Science.

[B45] Gui GP, Thomas PR, Tizard ML, Lake J, Sanderson JD, Hermon-Taylor J (1997). Two-year-outcomes analysis of Crohn's disease treated with rifabutin and macrolide antibiotics. J Antimicrob Chemother.

[B46] Borody TJ, Leis S, Warren EF, Surace R (2002). Treatment of severe Crohn's disease using antimycobacterial triple therapy – approaching a cure?. Dig Liver Dis.

[B47] Shafran I, Kugler L, El-Zaatari FA, Naser SA, Sandoval J (2002). Open clinical trial of rifabutin and clarithromycin therapy in Crohn's disease. Dig Liver Dis.

[B48] Borody TJ, Bilkey S, Wettstein AR, Leis S, Pang G, Tye S (2007). Anti-mycobacterial therapy in Crohn's disease heals mucosa with longitudinal scars. Dig Liver Dis.

[B49] Selby W, Pavli P, Crotty B, Florin T, Radford-Smith G, Gibson P, Mitchell B, Connell W, Read R, Merrett M, Ee H, Hetzel D, Antibiotics in Crohn's Disease Study Group (2007). Two-year combination antibiotic therapy with clarithromycin, rifabutin, and clofazimine for Crohn's disease. Gastroenterology.

[B50] Behr MA, Hanley J (2008). Antimycobacterial therapy for Crohn's disease: a reanalysis. Lancet Infect Dis.

[B51] Behr MA, Kapur V (2008). The evidence for *Mycobacterium paratuberculosis *in Crohn's disease. Curr Opin Gastroenterol.

[B52] Chamberlin W, Ghobrial G, Chehtane M, Naser SA (2007). Successful treatment of a Crohn's disease patient infected with bacteremic *Mycobacterium paratuberculosis*. Am J Gastroenterol.

[B53] Greenstein RJ, Su L, Haroutunian V, Shahidi A, Brown ST (2007). On the action of methotrexate and 6-mercaptopurine on *M. avium *subspecies *paratuberculosis*. PLoS One.

[B54] Greenstein RJ, Su L, Shahidi A, Brown ST (2007). On the action of 5-amino-salicylic acid and sulfapyridine on *M. avium including *subspecies *paratuberculosis*. PLoS One.

[B55] Shin SJ, Collins MT (2008). Thiopurine drugs azathioprine and 6-mercaptopurine inhibit *Mycobacterium paratuberculosis *growth in vitro. Antimicrob Agents Chemother.

[B56] Bryk R, Gold B, Venugopal A, Singh J, Samy R, Pupek K, Cao H, Popescu C, Gurney M, Hotha S, Cherian J, Rhee K, Ly L, Converse PJ, Ehrt S, Vandal O, Jiang X, Schneider J, Lin G, Nathan C (2008). Selective killing of nonreplicating mycobacteria. Cell Host Microbe.

